# Zinc Overload in Microvessels Contributes to Blood–Brain Barrier Disruption by Activating the JAK2 Pathway After Cerebral Ischemia/Reperfusion

**DOI:** 10.1002/cns.70885

**Published:** 2026-04-16

**Authors:** Zhengran Guo, Wenjuan Shi, Xiaodong Chen, Shuhua Yuan, Xunming Ji, Zhifeng Qi

**Affiliations:** ^1^ Department of Neurology, Cerebrovascular Diseases Research Institute Xuanwu Hospital of Capital Medical University Beijing China; ^2^ Department of Hyperbaric Oxygen, Beijing Chaoyang Hospital Capital Medical University Beijing China

**Keywords:** blood–brain barrier, inflammation, ischemia/reperfusion (I/R), JAK2/STAT3, microvessels, stroke, zinc, zinc transporter 3 (ZnT3)

## Abstract

**Aims:**

Stroke is one of the leading causes of adult disability and death worldwide. Inflammation‐induced microvascular dysfunction and increased blood–brain barrier (BBB) permeability are major contributors to cerebral ischemia/reperfusion (I/R) injury. Previous studies have shown that zinc accumulation in microvessels contributes to BBB disruption following I/R. However, the mechanisms linking zinc accumulation to microvascular inflammation remain poorly understood.

**Methods:**

We investigated whether the Janus kinase 2/signal transducer and activator of transcription 3 (JAK2/STAT3) inflammatory pathway mediates microvascular zinc overload‐induced BBB damage, using the model of I/R rats, endothelial cells and neuron‐specific zinc transporter 3 knockout (ZnT3‐cKO) mice.

**Results:**

Our findings in I/R rats and endothelial cells revealed that zinc accumulation in microvessels activated JAK2, promoting mitochondrial translocation of phosphorylated STAT3 (p‐STAT3) and exacerbating BBB disruption. These effects were significantly suppressed by zinc chelation. Furthermore, inhibition of neuronal zinc release in ZnT3‐cKO mice markedly reduced zinc accumulation and JAK2 activation in ischemic microvessels. ZnT3 knockout also prevented mitochondrial translocation of p‐STAT3, attenuated mitochondrial dysfunctions, and abolished zinc overload‐induced BBB permeability following I/R.

**Conclusion:**

This study suggests that zinc accumulation in microvessels contributes to I/R‐induced BBB damage through JAK2/STAT3 signaling and highlights a potential therapeutic target for preserving vascular integrity after stroke.

## Graphical abstract

Cerebral ischemia/reperfusion (I/R) induces zinc accumulation in ischemic microvessels, activating Janus kinase 2/signal transducer (JAK2). This promotes mitochondrial translocation of phosphorylated STAT3 (p‐STAT3), leading to mitochondrial dysfunction that exacerbates endothelial cell death and disrupts blood–brain barrier (BBB). This pathway is significantly suppressed by zinc chelation or neuron‐specific knockout of zinc transporter 3 (ZnT3‐cKO). These findings suggest a potential therapeutic target for protecting BBB after I/R.

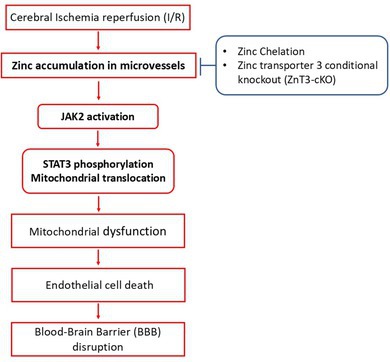

## 1 Introduction

Stroke is the second leading cause of adult disability and death worldwide in recent years, responsible for 5.9 million deaths each year [1]. Among the available treatments, recanalization therapies—including thrombolysis and mechanical thrombectomy—remain the most effective for restoring cerebral blood flow following ischemic stroke [2]. However, despite timely recanalization, over 50% of patients fail to achieve favorable outcomes [3]. This discrepancy suggests that factors beyond the restoration of blood flow contribute to poor prognosis after stroke [4].

Recent studies have highlighted inflammation‐induced microvascular dysfunction and increased blood–brain barrier (BBB) permeability as major contributors to ischemia/reperfusion injury [5, 6, 7, 8]. Disruption of the BBB not only exacerbates edema and hemorrhagic transformation but also amplifies neuroinflammation, further impairing recovery. Zinc, a trace element highly concentrated in the brain, has emerged as a key player in the pathogenesis of ischemic injury. While essential for normal neuronal function, zinc becomes neurotoxic when its extracellular levels rise sharply during cerebral ischemia, leading to neuronal apoptosis [9].

Our previous research demonstrated that excessive zinc accumulation in cerebral microvessels significantly contributes to cell death following ischemic stroke [7]. Zinc overload in the microvasculature also induces mitochondrial damage in endothelial cells, disrupting mitochondrial membrane potential and triggering apoptotic signaling [10]. Furthermore, neuron‐specific knockout of the zinc transporter 3 (ZnT3)—responsible for synaptic zinc release—reduced extracellular zinc levels and preserved BBB integrity in stroke models [11]. These findings highlight the pathological role of zinc overload. However, the mechanisms linking zinc accumulation to microvascular inflammation remain poorly understood.

JAK2/STAT3 signaling cascade, an important inflammatory pathway, is rapidly activated after ischemic stroke and promotes inflammation, oxidative stress, and apoptosis [12]. Importantly, inhibition of JAK2/STAT3 has been shown to alleviate BBB disruption and improve outcomes in experimental models [13]. This suggests a potential mechanistic link between zinc overload and JAK2/STAT3‐mediated vascular injury.

Based on these insights, we hypothesize that zinc accumulation in the microvasculature activates the JAK2/STAT3 inflammatory pathway, contributing to BBB disruption following ischemia/reperfusion. In this study, we investigated this hypothesis using a rat model of ischemic stroke and ZnT3 conditional knockout (ZnT3‐cKO) mice, aiming to elucidate the molecular mechanisms by which zinc overload exacerbates microvascular injury.

## 2 Materials & Methods

### 2.1 Animals

All animal experiments were approved by the Institutional Animal Care and Use Committee of Capital Medical University (Beijing, China). Group assignments, surgical procedures, and outcome assessments were conducted blindly by the experimental investigators. Animals were randomly allocated into groups using a random number table. Sprague–Dawley rats and C57 control mice were purchased from Beijing Vital River Laboratory Animal Co. Ltd. Transgenic mice with neuron‐specific knockout of ZnT3 (Slc30a3 fl/fl; Syn1‐Cre/+)—referred to as ZnT3‐cKO—were generated at Beijing Biocytogen Co. Ltd., as previously described. The littermates (Slc30a3fl/fl;+/+) of ZnT3‐cKO mice were used as control [11].

All animal procedures were approved (No. AEEI‐2024‐112) by the Institutional Animal Care and Use Committee of Capital Medical University, Beijing, China according to ARRIVE guidelines.

### 2.2 Transient Focal Cerebral Ischemia Model

Transient focal cerebral ischemia was induced in male SD rats (8 weeks old, 290–320 g) or transgenic mice (8 weeks old, 20–23 g) via middle cerebral artery occlusion (MCAO). Male animals were used in the present study to ensure consistency in testing our mechanistic hypotheses and to facilitate direct comparisons with existing literature where the majority of studies have been conducted in male rodents.

The MCAO surgery was conducted using a silicone‐coated filament (Cat: 403956PK5RE for rats, 602156PK5Re for mice, Doccol, USA) under 2% isoflurane anesthesia, as previously described [11]. Occlusion was maintained for 90 min in rats and 60 min in mice, followed by 24 h of reperfusion. Sham‐operated animals underwent the same surgical procedures without suture insertion.

### 2.3 Drug Administration

To investigate the role of zinc in BBB disruption following ischemia/reperfusion, rats were administered a specific zinc chelator, N,N,N′,N′‐tetrakis (2‐pyridylmethyl) ethylenediamine (TPEN). TPEN (15 mg/kg, Sigma‐Aldrich, MO, USA) or vehicle (10% dimethylsulfoxide in saline) was administered via intraperitoneal injection immediately after reperfusion, as described in our previous study [14].

To further explore the role of neuronal zinc on the JAK2 pathway and BBB injury, zinc chloride (ZnCl_2_) was administered to ZnT3‐cKO mice to restore extracellular free zinc levels during cerebral ischemia, based on our previous study [11]. ZnCl_2_ (60 nmol dissolved in 2 μL saline, Cat: Z0152, Sigma‐Aldrich) or equivalent volume saline was microinjected into the lateral ventricle (coordinates: 0.5 mm posterior to bregma, 1.5 mm lateral to the midline, and 2.5 mm below the surface of the brain) 30 min prior to MCAO.

### 2.4 Isolation of Cerebral Microvessels From the Brain of Ischemic Animals

**FIGURE 1:**
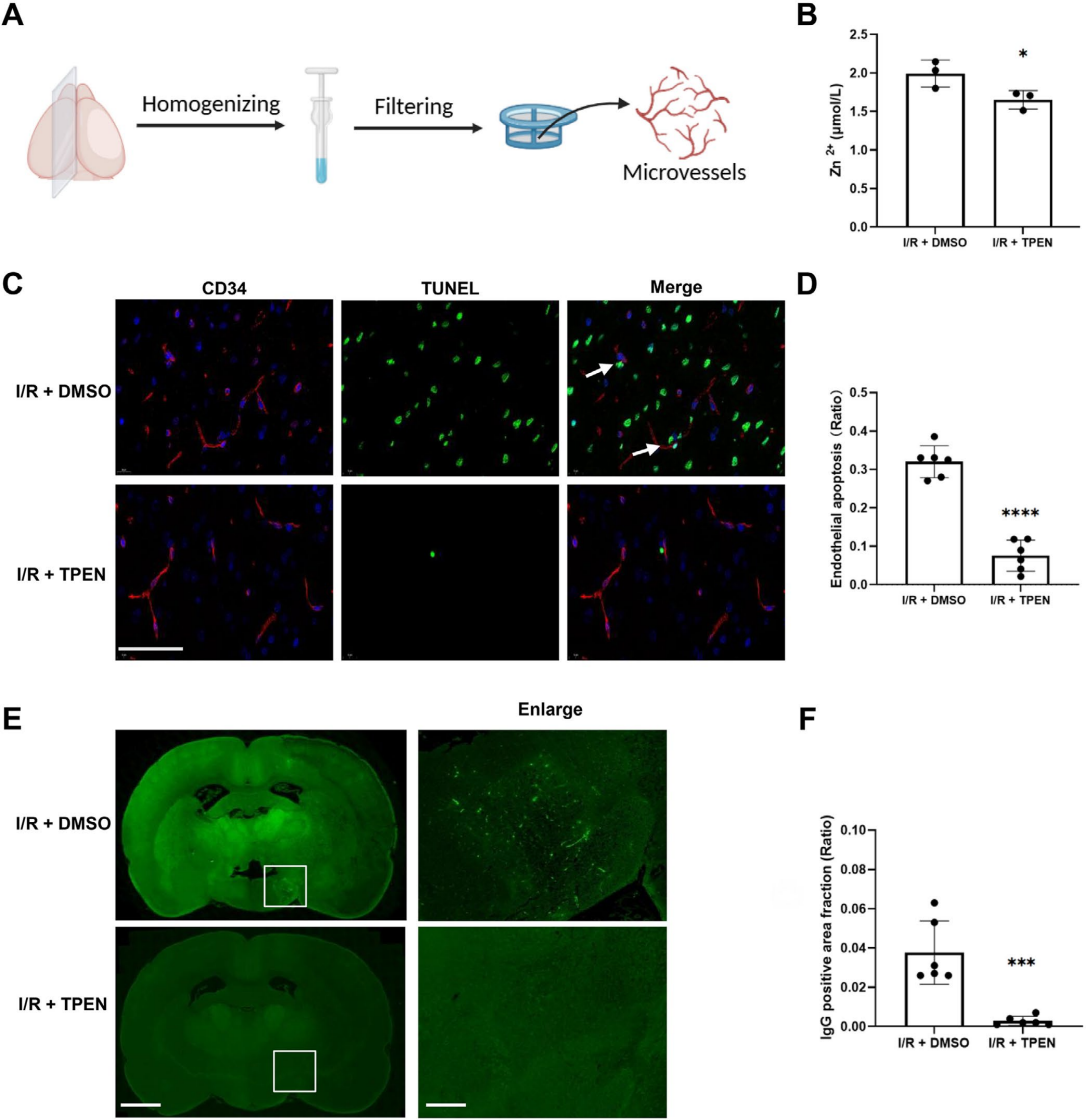
Zinc overload in cerebral microvessels exacerbated endothelial cell death and BBB disruption following cerebral I/R. (A) The schematic diagram of cerebral microvessels isolation. Ischemic brain tissue was collected from I/R rats after 90‐min ischemia followed by 24‐h reperfusion. Cerebral microvessels were isolated through tissue homogenization and filtration. (B) Zinc levels in cerebral microvessels were measured using a colorimetric assay in I/R rats treated with DMSO (vehicle) or TPEN (a specific zinc chelator, 15 mg/kg, intraperitoneally administered immediately after removing suture, *n* = 3 per group). (C, D) Representative images of CD34 (a microvessel‐specific marker, red) and TUNEL (apoptotic marker, green) co‐staining on brain sections from I/R rats with or without TPEN treatment (*n* = 6 per group). Scale bar: 20 μm (E, F) Immunohistochemical detection of endogenous IgG leakage in brain sections from I/R rats with or without TPEN treatment. Enlarged images showed regions outlined by white rectangles (*n* = 6 per group). Scale bars: 1 mm and 200 μm (enlarged). Data were presented as mean ± SEM. *p < 0.05, ***p < 0.001, ****p < 0.0001 vs. I/*R* + DMSO group.

To investigate the effects of ischemia‐induced zinc release on the cerebral microvessels, we isolated microvessels from brain tissue following cerebral ischemia/reperfusion, as described in our previous study [15]. Briefly, at the end of 24 h of reperfusion, cerebral microvessels were extracted from the ischemic hemisphere. The hemispheric brain tissue was dissected, homogenized in ice‐cold PBS and then filtered through 40 μm nylon mesh. Microvessels retained on the filters were purified using Dextran T‐500 and stored at −80°C for subsequent analysis. The diagram of isolating cerebral microvessels was shown in Figure 1A.

### 2.5 Isolation of Brain Mitochondria

To examine the levels of phosphorylated STAT3 (p‐STAT3) and Cytochrome C (Cyt C) in mitochondria during ischemia, high‐purity mitochondria were isolated from brain tissues using the Qproteome mitochondrial isolation kit (Qiagen, cat. 37612), as reported in our previous study [10]. The isolated mitochondrial and cytoplasmic fractions were stored at −80°C for Western blotting analysis.

### 2.6 Measurement of Mitochondrial Membrane Potential

Mitochondrial membrane potential (ΔΨm) was assessed using a JC‐1 mitochondrial membrane potential assay kit (Beyotime, China). Briefly, isolated mitochondria (10 μL, containing ~50 μg of mitochondrial protein) were incubated with JC‐1 working solution at 37°C for 10 min. Fluorescence was measured at 490/530 nm (green) and 525/590 nm (red). The mitochondrial membrane potential was quantified by calculating the ratio of red/green [16].

### 2.7 Detection of Mitochondrial Reactive Oxygen Species (ROS)

Mitochondrial superoxide levels were detected using the MitoSOX Red fluorescent probe (MedChemExpress) [17]. Briefly, purified mitochondrial (50 μg) isolated from the ischemia hemisphere was stained with MitoSOX Red (at a final concentration of 5 μM) at 37°C for 30 mins. Fluorescence intensity at 510/580 nm was normalized to the mean fluorescence in the corresponding control group.

### 2.8 Measurement of Zinc Content in Isolated Cerebral Microvessels

The isolated cerebral microvessels were homogenized to measure zinc concentration in microvessels using a zinc colorimetric assay kit (Elabscience Biotechnology, Wuhan, China). Optical density (OD) values were measured at 560 nm using a microplate reader (Thermo Fisher, MA, USA).

### 2.9 Zinc Staining in Isolated Cerebral Microvessels

Fresh isolated microvessels from mice were mounted on slides and stained with the zinc‐selective fluorescent indicator Newport Green (NG; Thermo Fisher Scientific, MA, USA) to visualize cytoplasmic free zinc. The slides were washed with PBS and incubated with NG (10 μM) in the dark for 30 min and then stained with DAPI (a nucleus‐specific marker). Fluorescent images were captured using a fluorescence microscope (Nikon 80i, Japan).

### 2.10 Western Blotting

Isolated microvessels or mitochondria were lysed in buffer containing 50 mM Tris–HCl (pH 7.6), 150 mM NaCl, 5 mM CaCl_2_, 0.05% Brij‐35, and 1% Triton X‐100 for 30 min, following protocols from our previous study. Protein samples were subjected to SDS‐PAGE and transferred to membranes. The blots were probed with primary antibodies: anti‐JAK2 (1:1000; Cell Signaling Technology), anti‐p‐JAK2 (1:200; Signalway Antibody), anti‐Cytochrome C (1:500; BD Biosciences), and anti‐p‐STAT3 (1:1000; CST). Anti‐β‐actin (1:1000; Thermo Fisher) or anti‐COX IV (1:500; Proteintech) served as loading controls. Protein expression levels were analyzed using ImageJ software and normalized to β‐actin or COX IV.

### 2.11 In Situ Immunohistochemistry Staining

At the end of 24‐h reperfusion, animals were transcardial perfused with ice‐cold PBS. Brain sections (3 mm thick, spanning from approximately 0 mm to −3.0 mm relative to bregma) were rapidly collected and fixed in 4% paraformaldehyde. Three consecutive sections (3 μm thick, at 1 mm intervals) were prepared for immunofluorescence staining, including assessments of IgG leakage, TUNEL staining, and p‐JAK2/CD34 co‐staining (see below). Images were captured using a fluorescence microscope.

#### 2.11.1 IgG Leakage

To evaluate BBB disruption following ischemic stroke, endogenous IgG leakage from blood into brain tissue was assessed [18]. Since IgG cannot be washed out by transcardial perfusion, its presence indicates BBB permeability. PFA‐fixed slides were stained with FITC‐labeled goat anti‐mouse IgG (1:400, cat. ZF‐0312, Zhongshan Jinqiao, China) or goat anti‐rat IgG (1:400, cat. ZF‐0315, Zhongshan Jinqiao, China), and signals were observed via fluorescence microscopy.

#### 2.11.2 TUNEL Staining

Endothelial cell death after 90‐min ischemia/24‐h reperfusion was measured using a TUNEL assay kit (Servicebio, Wuhan, China). Brain slides were co‐stained with CD34 (a specific endothelial cell marker, 1:2000, Servicebio, Wuhan, China) and DAPI for imaging capture. TUNEL and immunofluorescence co‐localization analysis was adapted from established double‐labeling protocols [19].

#### 2.11.3 p‐JAK2/CD34 Co‐Staining

Expression of p‐JAK2 in brain microvessels was assessed through co‐staining of p‐JAK2 (1:3000, Servicebio, Wuhan, China), CD34, and DAPI.

### 2.12 Statistical Analysis

All data were analyzed using the IBM SPSS Statistics for Windows version 26.0 (IBM Corp, Armonk, NY, USA). Statistical analyzes were performed using GraphPad Prism 9.4.0 (GraphPad Software Inc., San Diego, CA, USA). Normal distribution was determined using the Kolmogorov Smirnov test. For comparisons involving more than two groups, one‐way or two‐way ANOVA followed by Tukey's or post hoc multiple‐comparison tests were applied, as appropriate. For two‐group comparisons, two‐tailed unpaired Student's *t*‐tests were used. A value of *p* < 0.05 was considered statistically significant.

## 3 Results

### 3.1 Zinc Overload in Cerebral Microvessels Exacerbated Endothelial Cell Death and BBB Disruption Following Cerebral Ischemia/Reperfusion

Cerebral microvessels are essential for maintaining BBB integrity. To investigate the effects of zinc overload on BBB disruption following ischemic stroke, we isolated cerebral microvessels from the ischemic hemisphere of rats and measured zinc content using a colorimetric assay after 90‐min ischemia and 24‐h reperfusion. As shown in Figure 1B, the application of the zinc chelator TPEN effectively mitigated zinc overload in the microvessels following cerebral ischemia/reperfusion (I/R).

Additionally, we performed co‐staining of TUNEL and CD34 (a microvessel‐specific marker) on rat brain sections to assess endothelial cell apoptosis in response to zinc overload after I/R (Figure 1C). Fluorescence imaging demonstrated that a substantial number of TUNEL‐positive cells (green) co‐localized with CD34‐positive microvessels (red), indicating severe endothelial apoptosis after ischemia/reperfusion. Importantly, the number of apoptotic endothelial cells was reduced when zinc accumulation was blocked by TPEN administration (Figure 1D).

To evaluate the effects of zinc overload‐induced BBB integrity post ischemia/reperfusion, brain slices were analyzed for endogenous IgG leakage. The results demonstrated significant IgG extravasation in the I/*R* + DMSO group, indicating BBB disruption following ischemia/reperfusion. However, chelation of zinc overload with TPEN markedly decreased IgG leakage after 90‐min ischemia and 24‐h reperfusion (Figure 1E,F).

These findings suggest that zinc overload in ischemic cerebral microvessels contributes to endothelial apoptosis and compromises BBB integrity in rats subjected to ischemia/reperfusion.

### 3.2 Chelating Zinc Suppressed the Level of p‐JAK2 on Microvessels of Ischemic Rats

**FIGURE 2:**
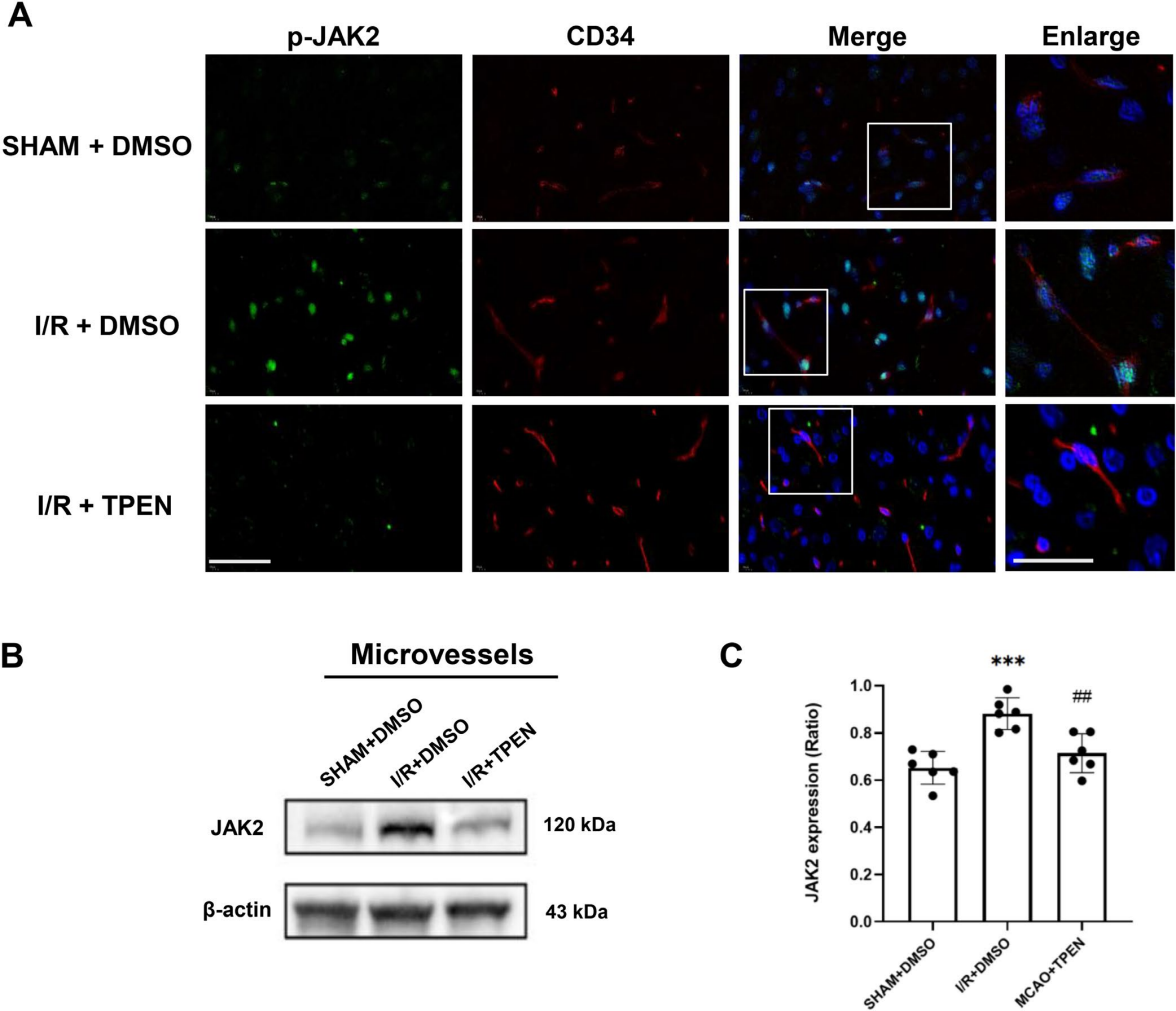
Chelating zinc suppressed the level of p‐JAK2 on microvessels of cerebral I/R rats after 90‐min ischemia/24‐h reperfusion. (A) Images of CD34 (red) and p‐JAK2 (green) staining of rat brain. Arrows indicate the co‐localization of CD34 and p‐JAK2 on microvessels. Enlarged images showed regions outlined by white rectangles. Scale bar: 20 μm and 10 μm (enlarge). (B, C) The level of JAK2 expression in microvessels from ischemic brain tissues of rats. Data were presented as mean ± SEM. ***p < 0.001 versus SHAM+DMSO group; ##*p* < 0.01 versus I/*R* + DMSO (*n* = 6 per group).

To investigate the role of JAK2 in zinc overload‐induced BBB disruption following ischemic stroke, we detected the level of total JAK2 and phosphorylated JAK2 (p‐JAK2, one of the active forms of JAK2) in cerebral microvessels after 90 min of ischemia and 24 h of reperfusion using the methods of in situ immunohistology and Western blot. Brain slides were co‐stained with p‐JAK2 (green) and CD34 (a specific endothelial cell marker, red). The results showed that the signal of positive p‐JAK2 was hardly seen in the microvessels of sham rats. The level of p‐JAK2 in microvessels was greatly elevated in I/*R* + DMSO group, which was inhibited by the administration of the zinc chelator, TPEN (Figure 2A).

Western blot was performed to further evaluate the JAK2 expression in isolated microvessels. The representative bands of Western blot (Figure 2B) and statistic data (Figure 2C) indicated that the level of JAK2 in microvessel was significantly elevated in the I/*R* + DMSO group, compared with the SHAM + DMSO group. Moreover, the administration of the zinc chelator TPEN to suppress zinc overload in microvessels prevented the level of JAK2 from elevation following ischemia/reperfusion.

We further determined whether zinc directly induces JAK2 phosphorylation using the mouse brain microvascular endothelial cell line (see Supporting Information, Figure S1).

These findings indicate that zinc overload elevates total JAK2 expression and facilitates its activation in microvessels, which may contribute to the microvascular damage observed after ischemic stroke.

### 3.3 Blocking Zinc Downregulated the Phosphorylation of STAT3 in Mitochondria and Abolished Mitochondrial Related Cell Damages in Ischemia/Reperfusion Rats

**FIGURE 3:**
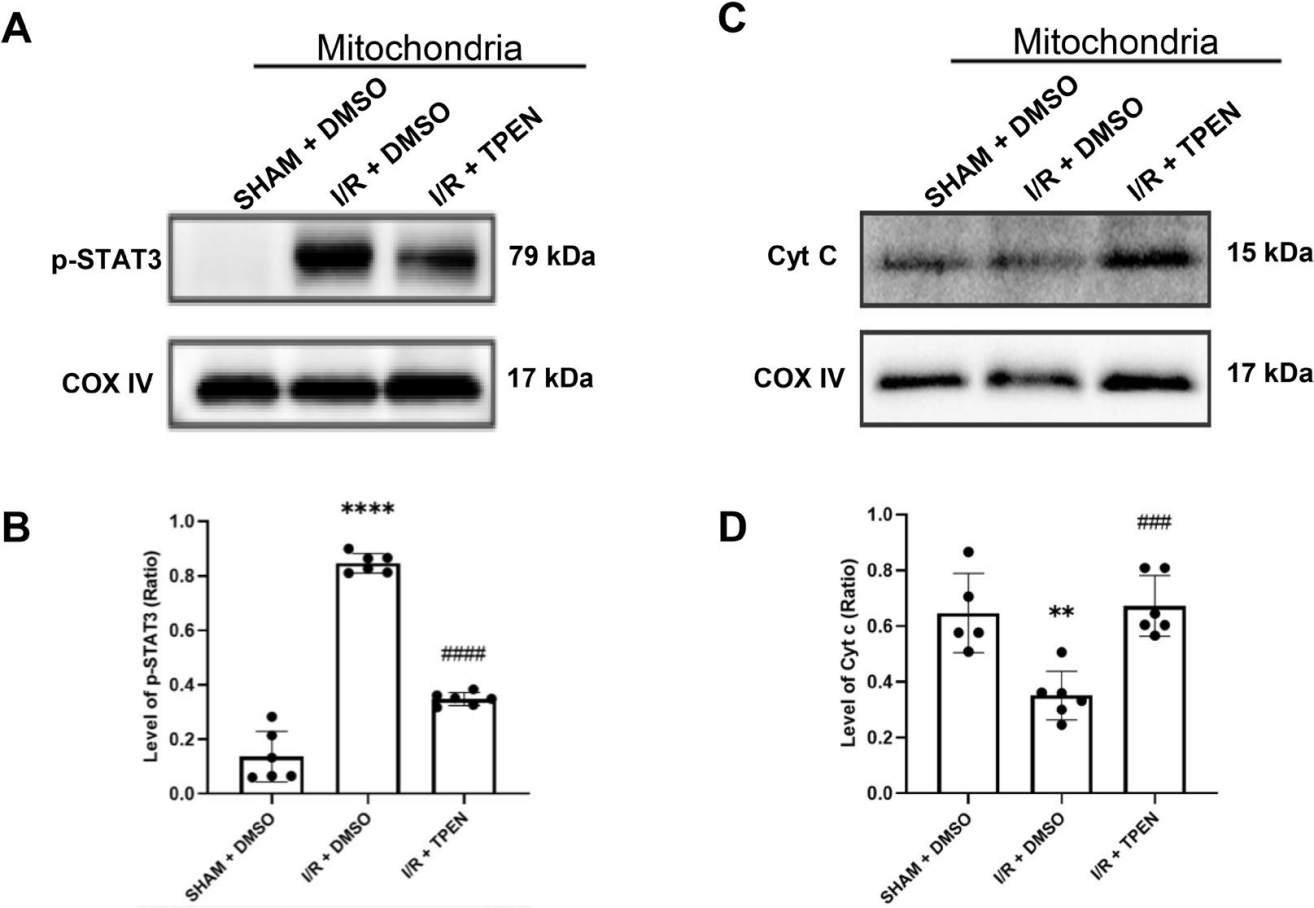
Blocking zinc downregulated the phosphorylation of STAT3 in mitochondria and abolished mitochondrial‐related cell damages in ischemia/reperfusion rats. (A, B) Western blot analysis of p‐STAT3 expression in isolated mitochondria fractions from the ischemic brain tissue of rats after 90‐min ischemia/24‐h reperfusion. (C, D) Western blot analysis of Cytochrome C (Cyt C) expression in isolated mitochondria. Data were presented as mean ± SEM. **p < 0.01, ****p < 0.0001 versus SHAM+DMSO, ###*p* < 0.001, ####*p* < 0.0001 versus I/*R* + DMSO (*n* = 6 per group).

Studies showed that the phosphorylated‐STAT3, one of the downstreams of JAK2, could translocate to mitochondria from cytosol and lead to mitochondrial damages following ischemia/reperfusion. Here, we isolated mitochondria from ischemic hemispheres and examined the mitochondrial translocation of p‐STAT3 in response to zinc overload after 90‐min ischemia and 24‐h reperfusion (Figure 3A,B).

The Western blot data showed that the level of p‐STAT3 in the mitochondrial fraction was pretty low in the SHAM + DMSO group, while ischemia/reperfusion significantly elevated the p‐STAT3 level in mitochondria. Furthermore, blocking zinc overload with TPEN administration significantly down‐regulated the level of p‐STAT3 in the mitochondria.

The effect of p‐STAT3 translocation on mitochondria was further evaluated by measuring cytochrome c release from mitochondria, which is crucial for the mitochondria‐dependent cell death pathway (Figure 3C,D). The Western blotting results showed that, compared to the SHAM + DMSO rats, the level of cytochrome c in mitochondria greatly declined in the I/*R* + DMSO group, while TPEN treatment significantly blocked the level of cytochrome c release from mitochondria after I/R.

These data suggested that as a downstream of JAK2, the p‐STAT3 translocation to mitochondria was involved in the zinc overload‐induced mitochondrial damage following ischemia/reperfusion.

### 3.4 Specific Knockout of Neuronal ZnT3 Prevented Zinc Overload and JAK2 Phosphorylation in Isolated Microvessels After Cerebral I/R

Our previous study showed that specifically knockout of neuronal zinc transporter ZnT3 prevents ischemia‐induced BBB disruption. Here we further investigate whether conditional knockout neuronal ZnT3 could relieve zinc overload‐induced JAK2 activation on microvessels following ischemia/reperfusion.

**FIGURE 4:**
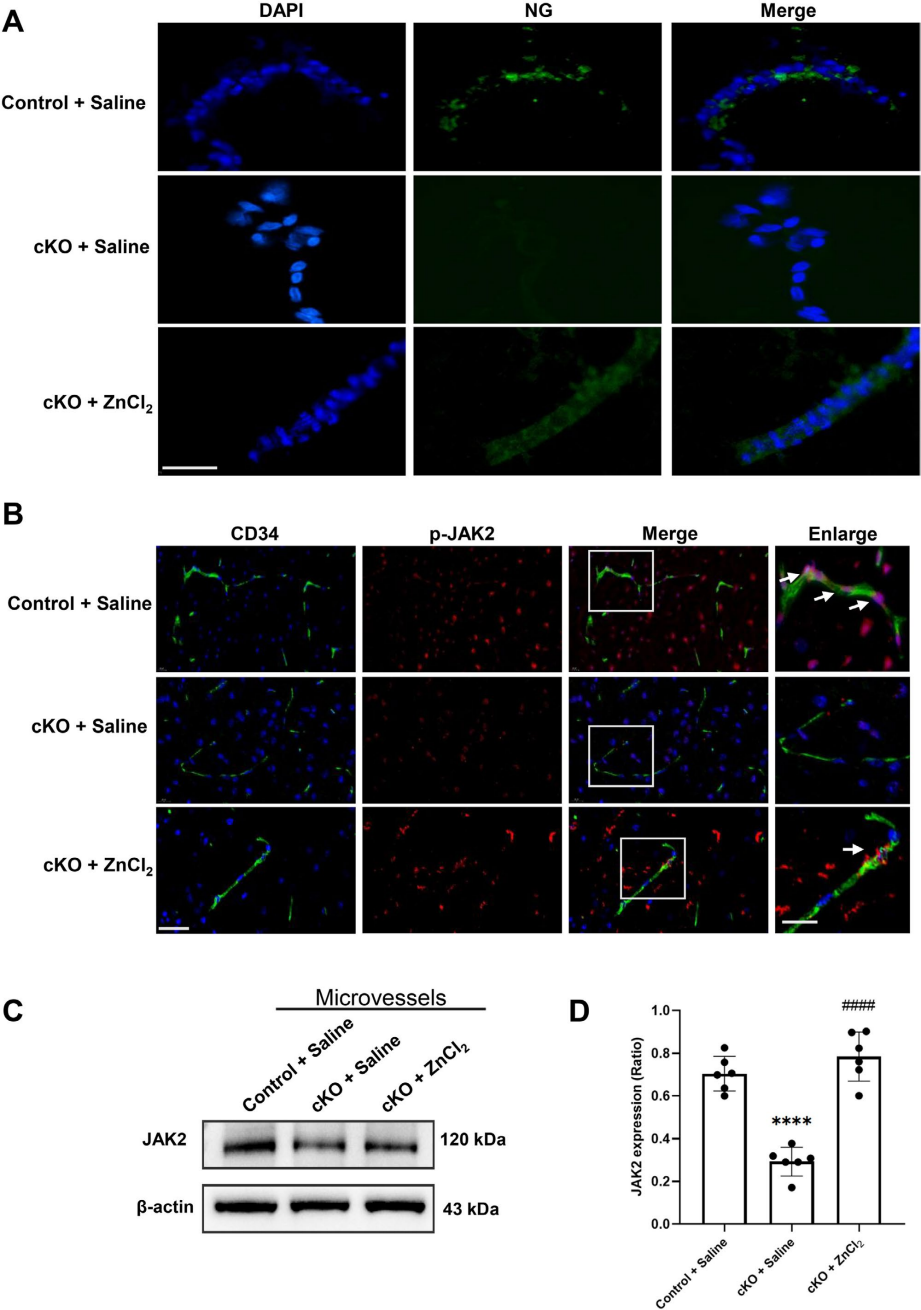
Specific knockout of neuronal ZnT3 prevented zinc overload and JAK2 phosphorylation in isolated microvessels tissue from mice with 60‐min ischemia/24‐h reperfusion. The microvessels were isolated from ZnT3‐cKO or control mice after 60‐min ischemia followed by 24‐h reperfusion. (A) The co‐stained with NG (a specific zinc indicator, green) and DAPI (blue) in isolated microvessels from the brain sections of ZnT3‐cKO and control mice, with or without ZnCl_2_ intracerebroventricular microinjection. Scale bar: 20 μm. (B) The co‐staining of CD34 (red) and p‐JAK2 (green) from brain tissues of ZnT3‐cKO or control mice. ZnCl_2_ (60 nmol in 2 μL saline) or saline was microinjected into the lateral ventricle of ZnT3‐cKO mice 30 min prior to MCAO to restore extracellular free zinc levels during cerebral ischemia. Enlarged images showed regions outlined by white rectangles. Arrows indicated the co‐localization of CD34 and p‐JAK2 on microvessels. Scale bar: 20 μm and 10 μm (enlarged). (C, D) The level of JAK2 expression in isolated microvessels from the ischemic brain. Data were presented as mean ± SEM. ****p < 0.0001 versus Control + Saline group, ####*p* < 0.0001 versus cKO + Saline group (*n* = 6 per group).

We isolated brain microvessels from the ischemic hemisphere of cKO or control mice and stained the isolated microvessels with NG staining (a specific zinc indicator) to measure the free zinc level in microvessels (Figure 4A). The fluorescent images demonstrated that there were bright NG positive signals (green) in isolated microvessels from control mice, showing zinc overload on microvessels after ischemia/reperfusion. In contrast, specific knockout ZnT3 could suppress the signal of NG positive staining on microvessels from the ischemic hemisphere. Furthermore, intracerebroventricular microinjection of ZnCl_2_ in ZnT3‐cKO mice significantly restored zinc levels in microvessels, as evidenced by increased NG‐positive fluorescence, highlighting the reversible effect of zinc supplementation on microvascular zinc accumulation.

To investigate whether conditional knockout neuronal ZnT3 could relieve zinc overload‐induced JAK2 activation on microvessels following ischemia/reperfusion, we examined the level of p‐JAK2 on microvessels in in situ brain slides of cKO or control mice using immunohistology (Figure 4B). The p‐JAK2 positive signals (green) were colocalized with CD34 (red) positive microvasculature in control mice following I/R. In contrast, specific knockout of ZnT3 to block zinc overload on microvessels could suppress the colocalization of p‐JAK2 with CD34 positive staining following I/R, which could be reversed by the pretreatment of ZnCl_2_ by lateral ventricle injection to ZnT3‐cKO mice to increase free zinc in the brain.

Western blot was performed to further measure the level of JAK2 expression in isolated microvessels (Figure 4C,D). The WB data showed that, compared to the Control + Saline group, neuronal‐specific knockout of ZnT3 significantly reduced the JAK2 expression in microvessels following I/R, which was rescued by ZnCl_2_ pretreatment.

These data suggested that specific knockout of neuronal ZnT3 prevented zinc overload and JAK2 activation on microvessels following ischemia/reperfusion.

### 3.5 Conditional Knockout of ZnT3 Inhibited p‐STAT3 Translation to Mitochondria and Suppressed Zinc Overload‐Induced Mitochondrial Apoptosis Following I/R

**FIGURE 5:**
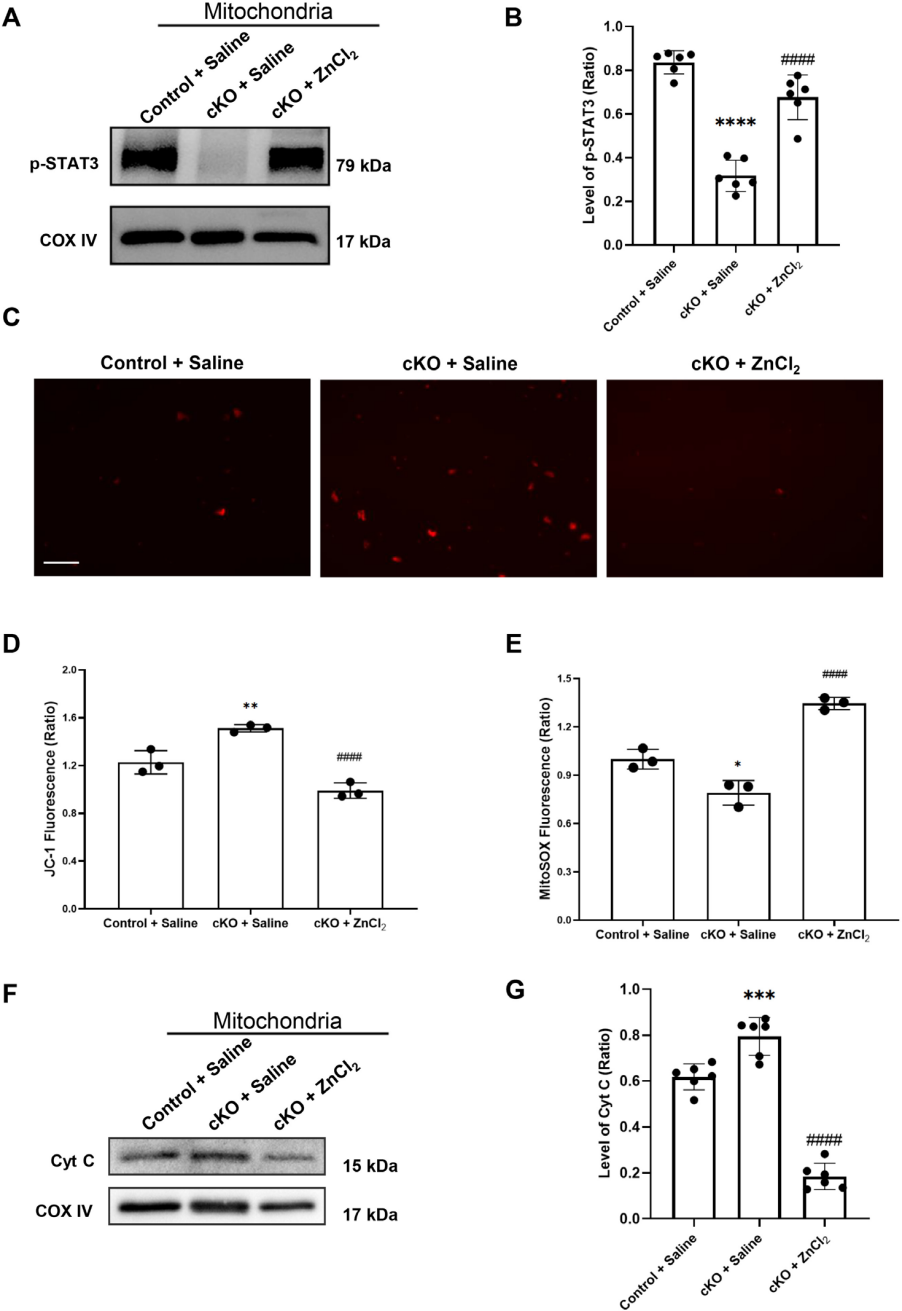
Conditional knockout of ZnT3 inhibited the phosphorylation of STAT3 in induced mitochondrial apoptosis in cerebral ischemia/reperfusion mice. (A, B) Western blot analysis of p‐STAT3 expression in isolated mitochondria from ZnT3‐cKO or control mice after 60‐min ischemia followed by 24‐h reperfusion (*n* = 6 per group). (C) Representative JC‐1 red fluorescence images showing mitochondrial membrane potential in mitochondria isolated from ischemic hemisphere. Scale bar: 100 μm. (D) Quantification of JC‐1 red/green fluorescence ratio in mitochondria isolated from ischemic hemisphere (*n* = 3 per group). (E) Quantitative analysis of mitochondrial ROS levels assessed by MitoSOX staining (*n* = 3 per group). (F, G) Western blot analysis of Cytochrome C (Cyt C) expression in brain mitochondria from ZnT3‐cKO or control mice with or without ZnCl_2_ treatment (*n* = 6 per group). Data were presented as mean ± SEM. *p < 0.05, **p < 0.01, ***p < 0.001, ****p < 0.0001 versus Control + Saline; ####*p* < 0.0001 versus cKO + Saline.

To further examine whether specific knockout of neuronal ZnT3 could suppress p‐STAT3 translocation to mitochondria and reduce zinc overload‐induced mitochondrial apoptosis after I/R, we isolated the mitochondria from the ischemic hemisphere of ZnT3‐cKO mice after 60‐min ischemia and 24‐h reperfusion. Western blot was performed to detect the level of p‐STAT3 in the mitochondria (Figure 5A,B). Compared to the Control + Saline group, neuronal specific knockout of ZnT3 greatly alleviated the mitochondrial translocation of p‐STAT3 following I/R. In contrast, the mitochondrial translocation of p‐STAT3 was significantly recovered with ZnCl_2_ pretreatment by lateral ventricle microinjection.

The mitochondrial membrane potential and oxidative stress were detected using JC‐1 staining and MitoSOX staining to evaluate mitochondrial dysfunction. JC‐1 staining showed that, compared to the Control + Saline group, ZnT3‐cKO mice exhibited a significantly higher red fluorescence intensity, which was markedly reversed by the ZnCl_2_ treatment via lateral ventricle microinjection (Figure 5C,D). MitoSOX staining demonstrated that neuronal ZnT3 knockout significantly decreased mitochondrial ROS generation after I/R, whereas intracerebroventricular ZnCl_2_ administration restored ROS production toward control levels (Figure 5E).

Furthermore, the levels of Cyt C in mitochondria from ischemic tissue of ZnT3‐cKO mice were more than those from the control mice, suggesting less Cyt C release from mitochondria to initiate mitochondria‐related cell apoptosis after I/R. Notably, the pretreatment with ZnCl_2_ into the lateral ventricle of ZnT3‐cKO mice greatly reversed the Cyt C release‐induced mitochondrial apoptosis following I/R (Figure 5F,G).

### 3.6 Conditional Knockout of ZnT3 Abolished Zinc Overload‐Induced Apoptosis on Microvessels and BBB Permeability Following Cerebral Ischemia/Reperfusion

**FIGURE 6:**
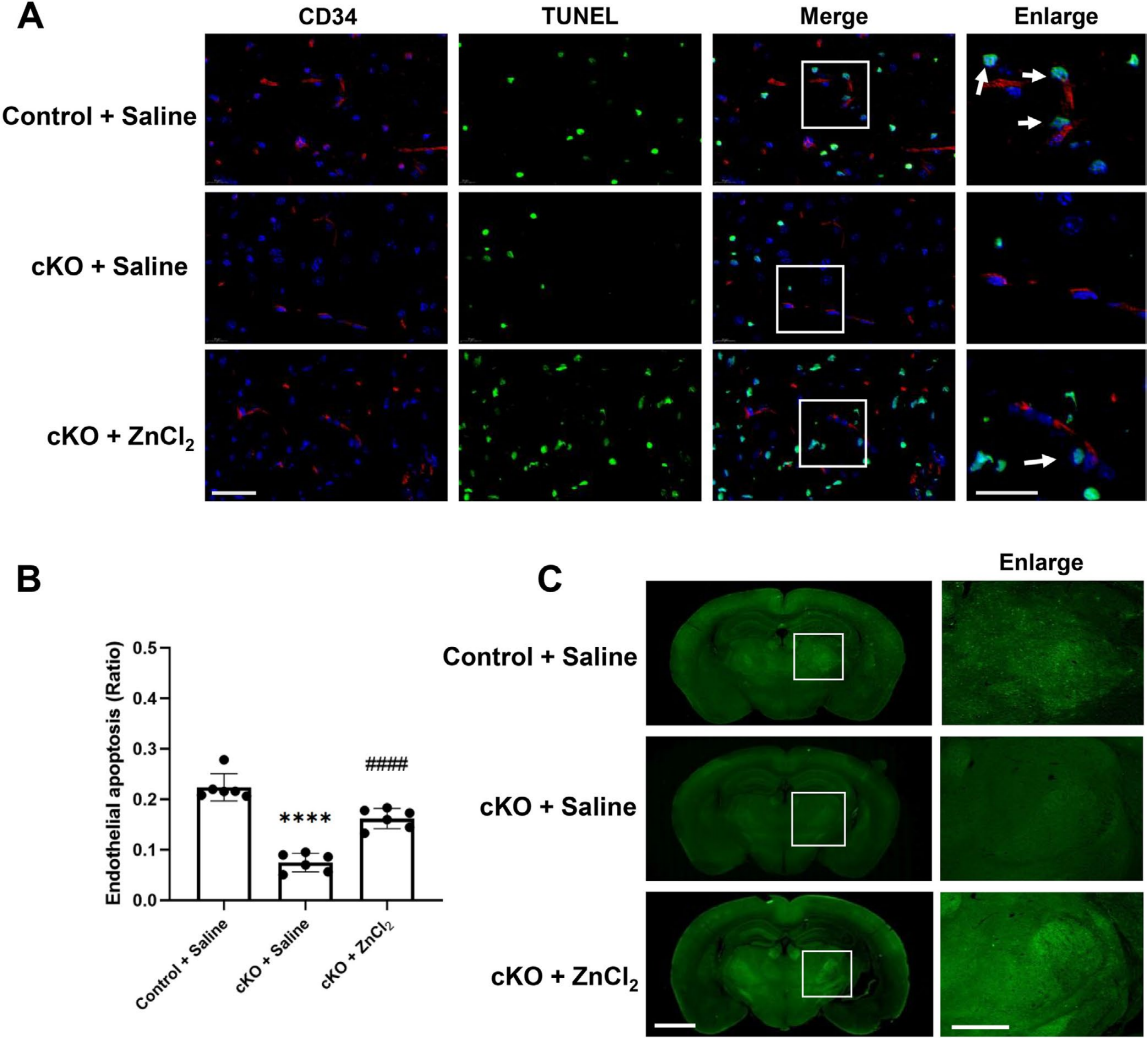
The elevation of zinc abolished ZnT3‐cKO induced on BBB protection following cerebral ischemia/reperfusion. The BBB leakage and endothelial cell death were measured in the ZnT3‐cKO or control mice after 60‐min ischemia followed by 24‐h reperfusion with or without ZnCl_2_ treatment. (A, B) The representative images and statistical chart of CD34 (a specific microvessel marker, red) and TUNEL (an apoptotic marker, green) co‐staining. Arrows indicated the co‐localization of CD34 and TUNEL on microvessels. Scale bar: 20 μm or 10 μm (enlarged). Data were presented as mean ± SEM. ****p < 0.0001, versus Control + Saline group, ####*p* < 0.0001 versus cKO + Saline group (*n* = 6 per group). (C) The representative images of endogenous IgG leakage measured using immunohistochemistr. Scale bar: 1 mm or 200 μm (enlarged). Enlarged images showed regions outlined by white rectangles.

Finally, we investigated the effects of conditional knockout of neural ZnT3 on zinc overload‐induced endothelial cell death and BBB disruption after I/R. The co‐staining of TUNEL and microvascular‐specific markers CD34 was applied to measure the apoptosis in microvasculature (Figure 6A,B). Compared to the Control + Saline group, there were less TUNEL positive cells colocalized with CD34 positive microvasculature in ZnT3‐cKO mice following I/R, suggesting less endothelial cell death after blocking zinc overload on microvessels. Moreover, microinjection of ZnCl_2_ into the lateral ventricle to supplement the zinc level could abolish the protective effects of ZnT3‐cKO on microvascular apoptosis.

We further measured the IgG permeability from the compromised BBB to the brain to assess the effects of ZnT3‐cKO on zinc overload‐induced BBB disruption after I/R (Figure 6C). Compared to the Control + Saline group, neuronal‐specific knockout of ZnT3 markedly blocked BBB disruption following I/R, which was severely rescued by pretreatment of ZnCl_2_ injection into the lateral ventricle and abolished the protective effect of ZnT3 knockout on the BBB following I/R.

## 4 Discussion

In this study, we report a novel mechanism underlying cerebral I/R‐induced BBB disruption that zinc accumulation in cerebral microvessels activates the JAK2/STAT3 signaling pathway, leading to mitochondria‐mediated cell death and subsequent BBB impairment in rat and neuronal‐specific ZnT3‐cKO mouse models.

Microvascular endothelial cells are a critical component of the BBB, and their integrity is essential for maintaining vascular homeostasis. Damage to these cells after ischemic stroke contributes significantly to BBB breakdown and inflammatory progression [20, 21], which are closely associated with poor clinical outcomes [22, 23]. While our previous work showed that elevated levels of zinc accumulation lead to endothelial cell death [7], the signaling mechanisms driving this process remained unclear. Identifying these pathways is key to developing effective therapies to protect the BBB after stroke.

Zinc, a metal ion abundantly distributed in the brain, plays complex roles in neurophysiology and neuropathology. While tightly regulated zinc signaling is essential for normal neuronal function, excessive accumulation of free zinc has been increasingly recognized as a critical mediator of neuronal toxicity and BBB dysfunction during I/R injury [24, 25].

Ischemic insult triggers excessive release of vesicular zinc from presynaptic terminals, while energy failure and oxidative stress impair intracellular zinc buffering systems and promote zinc release from metallothioneins and other zinc‐binding proteins [26]. During reperfusion, mitochondrial dysfunction, reactive oxygen species production, and blood–brain barrier disruption further exacerbate abnormal zinc redistribution within the neurovascular unit, leading to pathological zinc accumulation in both neuronal and vascular compartments [27]. Our prior studies established that zinc overload contributes to increased BBB permeability after stroke [28]. Moreover, zinc accumulation after reperfusion induces pro‐inflammatory signaling, highlighting its role as a trigger for post‐ischemic inflammation [29]. Recent studies further suggest that restoring zinc homeostasis after I/R can alleviate damage to the neurovascular unit [30]. However, direct zinc chelation has been proposed as a potential therapeutic strategy; its clinical translation is limited by the toxicity and poor specificity of currently available chelators, such as TPEN. Therefore, elucidating the mechanisms underlying zinc accumulation–induced injury may help identify more effective and safer therapeutic targets to protect against BBB disruption caused by zinc overload.

The present study shows that zinc overload specifically in cerebral microvessels is a key trigger for endothelial injury via the JAK2/STAT3 pathway. We found that cerebral I/R led to increased zinc accumulation in microvessels, accompanied by elevated JAK2 expression and phosphorylation. Chelation of zinc using TPEN effectively reduced zinc levels and suppressed JAK2 activation, resulting in decreased mitochondria‐mediated endothelial death and improved BBB integrity.

Our previous study indicated that specific knockout of neuronal ZnT3 can effectively inhibit the release of zinc from neurons and weaken the role of zinc in ischemia‐induced blood–brain barrier damage. To further validate this mechanism, we employed ZnT3‐cKO mice in the present study to investigate the role of zinc overload in vascular endothelial damage. The results in the present study showed that genetic deletion of ZnT3 reduced vascular zinc levels, inhibited JAK2 activation, and restored BBB function following I/R. These findings indicate that zinc accumulation is an upstream event that promotes activation of the JAK2/STAT3 pathway in microvascular endothelial cells.

JAK2/STAT3 signaling has previously been implicated in endothelial inflammation in various disease contexts. Beyond its canonical transcriptional role, STAT3 can localize to mitochondria and regulate mitochondrial respiration and redox homeostasis [31, 32]. However, its role in endothelial mitochondrial dysfunction under ischemic conditions remains unclear.

Zinc dyshomeostasis is increasingly recognized as a key driver of mitochondrial injury following cerebral ischemia. Excess zinc disrupts the mitochondrial membrane potential, enhances ROS production, and promotes mitochondrial permeability transition and cytochrome c release [33, 34]. Consistent with this, our previous work demonstrated that mitochondrial zinc overload increases ROS levels and upregulates Dynamin‐related protein 1 (DRP1) and Matrix metalloproteinase‐2 (MMP‐2), leading to mitochondrial fragmentation and BBB disruption [10, 27]. Despite these findings, it remains unclear whether JAK2/STAT3 signaling links zinc accumulation to mitochondrial dysfunction. Here, we show that zinc activates JAK2/STAT3 signaling and promotes STAT3 translocation to mitochondria, thereby exacerbating mitochondrial damage and endothelial injury following I/R.

Our result demonstrates that mitochondrial translocation of p‐STAT3 was associated with significant mitochondrial damage and apoptosis. Notably, zinc chelation or ZnT3‐cKO attenuated mitochondrial p‐STAT3 levels and mitochondrial injury, confirming the link between zinc, JAK2/STAT3 activation, and mitochondrial dysfunction (lower mitochondrial membrane potential and mitochondrial ROS generation). Conversely, supplementation with ZnCl_2_ restored zinc levels and reactivated this pathological cascade.

These findings suggest that the JAK2/STAT3 pathway acts as a zinc‐sensitive sensor in cerebral microvascular endothelial cells, mediating mitochondria‐related cell death that ultimately compromises the BBB. Our study provides the first evidence that mitochondrial translocation of STAT3—triggered by zinc‐induced JAK2 activation—plays a central role in BBB disruption post‐ischemia.

JAK2 inhibitors are already clinically approved for other conditions, such as myeloproliferative disorders and inflammatory skin diseases, and have shown favorable safety profiles [35, 36]. Based on our results, targeting JAK2 in the early stages of reperfusion may offer a promising strategy for preserving microvascular integrity and improving stroke outcomes. Taken together, these findings suggest that elucidating the downstream pathological mechanisms of zinc accumulation, particularly the JAK2/STAT3 signaling pathway, may provide a translationally relevant foundation for developing therapeutic strategies aimed at the prevention and early intervention of ischemic stroke.

## 5 Conclusion

Our data reveal a previously unrecognized mechanism in which post‐ischemic zinc accumulation in microvessels activates the JAK2/STAT3 pathway, promotes mitochondrial dysfunction in endothelial cells, and exacerbates BBB injury. These insights offer new therapeutic targets for protecting vascular integrity following stroke and may inform future strategies to improve patient outcomes after reperfusion therapy.

## Author Contributions

Zhengran Guo conceived and designed the experiments; performed the experiments; wrote the manuscript. Wenjuan Shi, Xiaodong Chen, Shuhua Yuan, and Xunming Ji analyzed and interpreted the data; Zhifeng Qi conceived and designed the experiments, contributed reagents, materials, analysis tools and data, and edited the manuscript.

## Funding

This work was supported in part by grants from the National Natural Science Foundation of China (82271308 to ZQ), the Beijing Chao‐Yang Hospital Golden Seeds Foundation (CYJZ202401 to SY), and the Beijing Natural Science Foundation (7262044 to SY).

## Ethics Statement

All animal procedures were approved (No. AEEI‐2024‐112) by the Institutional Animal Care and Use Committee of Capital Medical University, Beijing, China according to ARRIVE guidelines.

## Conflicts of Interest

The authors declare no conflicts of interest.

## Supporting information


**Figure S1:** Zinc induced JAK2 phosphorylation in cultured brain microvascular endothelial cells. (A, B) Representative image and analysis of p‐JAK2 levels in bEnd.3 cells treated with ZnCl_2_ under normoxic conditions (*n* = 6 per group). (C) ELISA‐based quantification of p‐JAK2 concentration under normoxia condition (*n* = 3 per group). (D, E) Representative image and analysis of p‐JAK2 expression in bEnd.3 cells subjected to oxygen glucose deprivation for 2 h followed by 24 h reoxygenation (OGD/R) in the presence of ZnCl_2_ (*n* = 6 per group). (F) ELISA‐based quantification of p‐JAK2 concentration under OGD/R conditions (*n* = 3 per group). Data are presented as mean ± SEM. *p < 0.05, **p < 0.01, ***p < 0.001 ****p < 0.0001.

**Supinfo 1.** cns70885‐sup‐0002‐Supplementarydata.docx.

## Data Availability

The data that support the findings of this study are available from the corresponding author upon reasonable request.
