# Supplementary figures and images for: Zinc Overload in Microvessels Contributes to Blood–Brain Barrier Disruption by Activating the JAK2 Pathway After Cerebral Ischemia/Reperfusion
Source: CNS Neurosci Ther. 2026 Apr 16;32(4):e70885. doi: 10.1002/cns.70885 (PMC13085896; doi:10.1002/cns.70885)

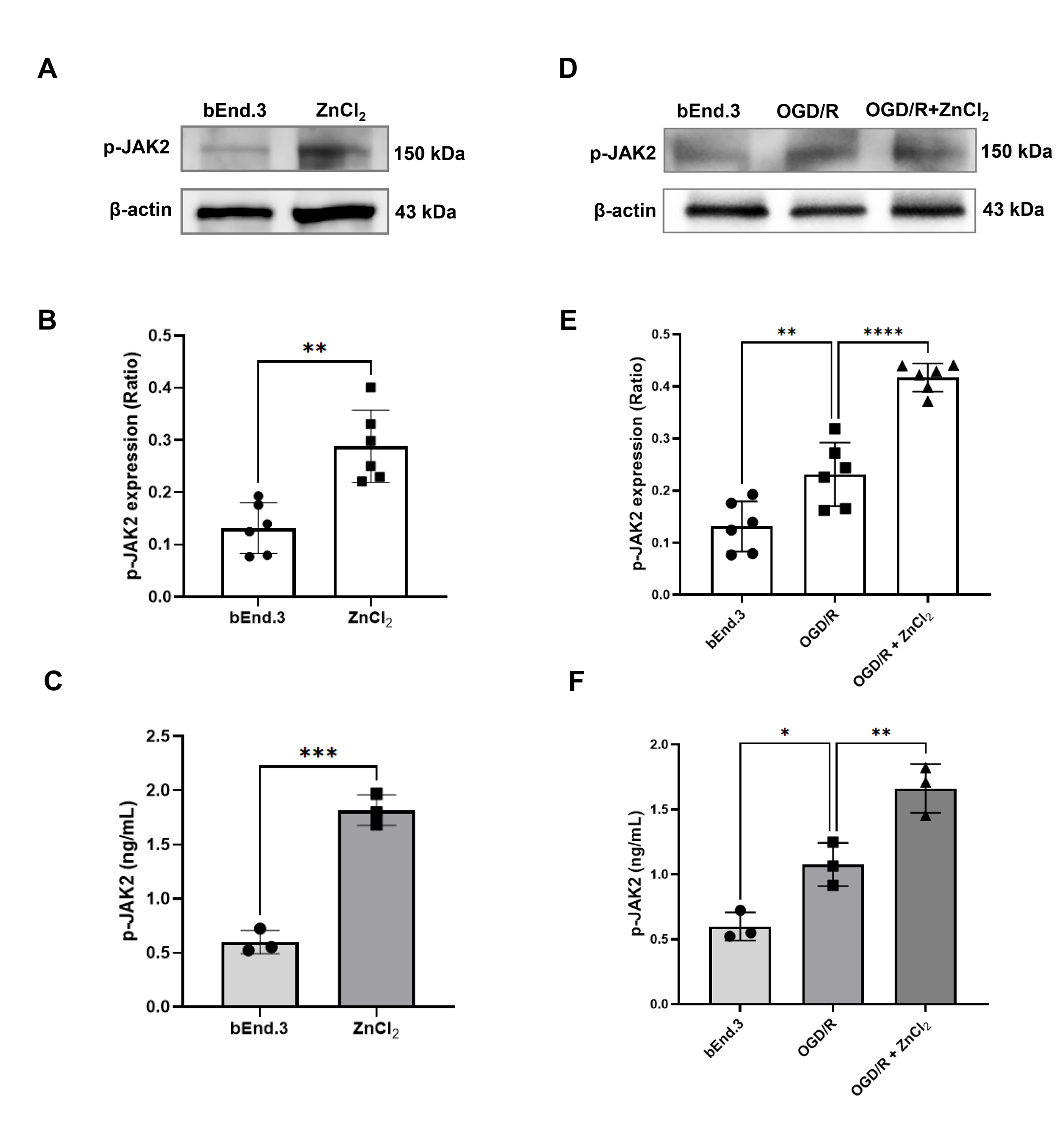

Supplement: Supplementary file 1 — Figure S1: Zinc induced JAK2 phosphorylation in cultured brain microvascular endothelial cells. (A, B) Representative image and analysis of p‐JAK2 levels in bEnd.3 cells treated with ZnCl2 under normoxic conditions (n = 6 per group). (C) ELISA‐based quantification of p‐JAK2 concentration under normoxia condition (n = 3 per group). (D, E) Representative image and analysis of p‐JAK2 expression in bEnd.3 cells subjected to oxygen glucose deprivation for 2 h followed by 24 h reoxygenation (OGD/R) in the presence of ZnCl2 (n = 6 per group). (F) ELISA‐based quantification of p‐JAK2 concentration under OGD/R conditions (n = 3 per group). Data are presented as mean ± SEM. *p < 0.05, **p < 0.01, ***p < 0.001 ****p < 0.0001. [file CNS-32-e70885-s001.tif]
