# Supplementary material for: Zinc Overload in Microvessels Contributes to Blood–Brain Barrier Disruption by Activating the JAK2 Pathway After Cerebral Ischemia/Reperfusion
Source: CNS Neurosci Ther. 2026 Apr 16;32(4):e70885. doi: 10.1002/cns.70885 (PMC13085896; doi:10.1002/cns.70885)
Supplement: Supplementary file 2 — Supinfo 1. cns70885‐sup‐0002‐Supplementarydata.docx. [file CNS-32-e70885-s002.docx]

**Supplementary data**

**Methods**

**Cell culture**

Mouse brain microvascular endothelial cells bEnd3 were obtained from Servicebio (Wuhan, China). Cells were maintained in Dulbecco's Modified Eagle Medium (DMEM) supplemented with 10% fetal bovine serum and 1% penicillin-streptomycin (Servicebio, Wuhan, China) at 37°C in a humidified incubator with 5% CO_2_, as previously described ^1^.

**Oxygen glucose deprivation/reoxygenation**

An *in vitro* oxygen–glucose deprivation/reoxygenation (OGD/R) model was established to mimic ischemia–reperfusion injury. Briefly, glucose-free DMEM (DMEM-G(-); Servicebio, Wuhan, China) was pre-equilibrated under hypoxic conditions (95% N_2_/5% CO_2_). bEnd.3 cells were then transferred into a modular hypoxia chamber flushed with the same gas mixture incubated at 37°C for 2 h. The oxygen concentration in the incubator was maintained below 0.2%. For reoxygenation, cells were removed from the hypoxic chamber, the medium was replaced with normal glucose-containing DMEM supplemented with 10% fetal bovine serum, and cultures were returned to normoxic conditions (37°C, 5% CO₂) for 24 h. Control cells were maintained in complete DMEM under normoxic conditions for the same duration, as previously described. For cell-based assays, bEnd.3 cells at 80–90% confluence were treated with ZnCl₂ (150 μM) or an equivalent volume of glucose-free DMEM or complete DMEM^1^.

**Measurement of p-JAK2 using ELISA**

Proteins were extracted from bEnd.3 cells using a lysis buffer containing protease and phosphatase inhibitors (Cat: 4693132001, 4906837001, Roche, Switzerland). The concentration of phosphorylated JAK2 (p-JAK2) was quantified using a mouse p-JAK2 Enzyme-Linkd Immunosorbent Assay (ELISA) kit (Enzyme-linked Biotechnology Co., Ltd., Shanghai, China), according to the manufacturer’s instructions. Absorbance at 450 nm was measured with a microplate reader (Thermo Fisher), and p-JAK2 levels were calculated from a standard curve^2^.

**Results**

**Zinc induced JAK2 phosphorylation in cultured brain microvascular endothelial cells**

To further determine whether zinc overload is sufficient to directly activate JAK2 signaling, we detected the level of JAK2 phosphorylation using Western blotting and ELISA in cultured cerebral microvascular endothelial cells (bEnd.3). Western blot data showed that under normoxic conditions, ZnCl₂ treatment (150 μM) resulted in a marked increase in JAK2 phosphorylation, compared to the control cells (**Fig. S1A, B).** The ELISA data also showed the similar results that JAK2 phosphorylation was activated by ZnCl₂ treatment (**Fig. S1C).**

We detected the JAK2 phosphorylation in responses to zinc overload in bEnd.3 cells followed by OGD-2h/ reoxygenation-24h. Western blotting data showed that ZnCl₂ exposure enhanced JAK2 phosphorylation (**Fig. S1D, E**). ELISA assay also showed that JAK2 phosphorylation were significantly enhanced by ZnCl₂ exposure (**Fig. S1F**).

These *in vitro* results provide direct mechanistic evidence that zinc accumulation triggers endothelial JAK2 activation, thereby strengthening the potential link between microvascular zinc overload and JAK2/STAT3 signaling observed *in vivo* after cerebral reperfusion.

**References**

1. Z Q, J L, R P, W D, J S, Y Y, et al. Zinc contributes to acute cerebral ischemia-induced blood-brain barrier disruption. *Neurobiology of disease*. 2016;95:12-21

2. Zhang C, Song J, Zhang W, Huang R, Li Y-J, Zhang Z, et al. Jak2/stat3 signaling in myeloid cells contributes to obesity-induced inflammation and insulin resistance. *Cells*. 2025;14
